# N^7^–SSPP Confers Drought Tolerance in *Arabidopsis*

**DOI:** 10.3390/ijms27062651

**Published:** 2026-03-13

**Authors:** Mengyuan Zhang, Kaixuan He, Xiaoyan Lv, Yujia Zhao, Yuanyuan Mei, Dan Wang, Ning Ning Wang

**Affiliations:** Tianjin Key Laboratory of Protein Sciences, Department of Plant Biology and Ecology, College of Life Sciences, Nankai University, Tianjin 300071, China; 1120190467@mail.nankai.edu.cn (M.Z.); hekaixuan@nankai.edu.cn (K.H.); 1120230692@mail.nankai.edu.cn (X.L.); 1120240807@mail.nankai.edu.cn (Y.Z.);

**Keywords:** SSPP, N^7^-element, protein degradation, ROS, drought tolerance

## Abstract

Drought tolerance is critical for plant survival and productivity and is tightly linked to redox homeostasis and senescence regulation. SENESCENCE-SUPPRESSED PROTEIN PHOSPHATASE (SSPP), a negative regulator of leaf senescence, has previously been implicated in salt stress tolerance. However, whether SSPP functions in drought stress responses remains unknown. Here, we demonstrate that SSPP enhances drought tolerance in *Arabidopsis thaliana*. Although drought represses *SSPP* transcription, drought treatment attenuated N^7^-mediated SSPP degradation, in which N^7^, the N-terminal 1–14 amino acids of AtACS7, functions as a conditional degradation signal, resulting in stress-responsive accumulation of SSPP protein in *N^7^-SSPP-overexpressing* plants. Both *SSPP-* and *N^7^-SSPP-overexpressing* plants exhibited enhanced drought tolerance, with survival rates after rewatering reaching approximately 95% and 70%, respectively, whereas the *sspp-1* mutant displayed pronounced drought sensitivity. Mechanistically, *SSPP* overexpression upregulated reactive oxygen species (ROS)-scavenging genes, enhanced antioxidant enzyme activities, and reduced drought-induced ROS accumulation, thereby mitigating oxidative damage. Notably, the N^7^ element enables conditional accumulation of SSPP under adverse conditions while preventing growth inhibition under normal conditions. Together, our findings reveal SSPP as a regulator connecting senescence-associated processes with drought stress adaptation and highlight the N^7^-SSPP fusion as a versatile strategy for improving stress resilience without compromising plant growth.

## 1. Introduction

Drought stress imposed by soil drying is one of the most predominant abiotic stresses limiting plant growth, productivity, and crop yield throughout the world [1,2]. With the increasing frequency and intensity of drought events under global climate change, improving drought tolerance while maintaining optimal growth and productivity has become a major goal of modern agriculture [3,4]. Leaf senescence is a genetically programmed and age-related active process that leads to chlorophyll degradation, reduced photosynthetic efficiency, and excessive accumulation of reactive oxygen species (ROS) [5,6]. Increasing evidence indicates that delayed leaf senescence is often associated with enhanced tolerance to adverse environmental conditions, including drought stress [7,8,9].

SENESCENCE-SUPPRESSED PROTEIN PHOSPHATASE (SSPP), a clade-D type 2C protein phosphatase, has been identified as an important negative regulator of leaf senescence [10]. We previously demonstrated that overexpression of *SSPP* delayed senescence progression and enhanced resistance to abiotic stresses by promoting ROS scavenging capacity. Notably, *SSPP* overexpression significantly improved salt stress tolerance and increased yield in soybean, highlighting its potential value for crop improvement [11]. These findings suggest that SSPP may function as a core regulatory node integrating senescence and environmental stress responses.

However, constitutive overexpression of *SSPP* severely inhibits vegetative growth, limiting its practical application [10]. We previously identified the N-terminal 1–14 amino acids of AtACS7, termed N^7^, as a conditional degradation signal that mediates ubiquitin/proteasome-dependent degradation of fused proteins [12]. Under normal conditions, the fusion of N^7^ to the N-terminus of SSPP promotes its degradation, alleviating SSPP-induced growth defects. In contrast, N^7^-mediated degradation is suppressed by specific developmental cues and environmental signals, including senescence and salt stress, enabling conditional accumulation of SSPP protein. Consequently, the N^7^-SSPP fusion largely restores normal growth while retaining the beneficial effects of SSPP on delaying leaf senescence and enhancing salt tolerance [11,12].

Despite these advances, whether SSPP contributes to drought tolerance and, importantly, whether drought stress engages the N^7^-mediated protein stability control system remain unknown. Unlike salt stress, soil-drying drought is typically a progressive and long-lasting stress that is tightly coupled to cellular dehydration, oxidative imbalance, and senescence progression [13]. These features make drought a distinct molecular context in which to test whether N^7^ functions as a general stress-responsive degradation signal or whether its regulation is stress-type specific. Thus, investigating SSPP and N^7^-mediated stability control under drought conditions provides an opportunity to determine how different environmental stresses converge on, or diverge from, a shared mechanism of protein regulation.

In this study, we demonstrated that SSPP positively regulates drought tolerance in *Arabidopsis*. We further showed that drought stress suppresses N^7^-mediated SSPP degradation at the protein level, thereby enabling stress-specific accumulation of SSPP. Our findings revealed that SSPP integrates drought-induced oxidative stress and senescence signaling to promote drought tolerance and highlight the potential of N^7^-SSPP as a conditional, stress-responsive strategy for improving crop resilience to drought and salinity stresses.

## 2. Results

### 2.1. SSPP Expression Is Down-Regulated in Response to Drought Stress

**Figure 1:**
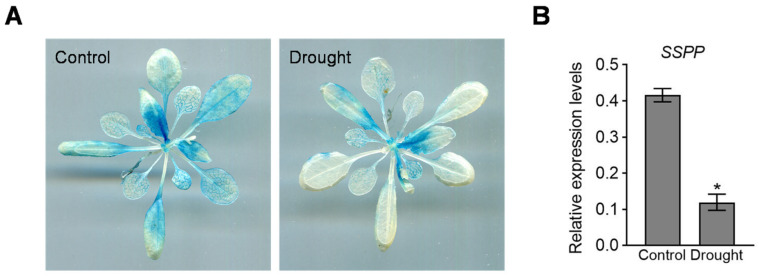
Drought stress down-regulates *SSPP* expression: (**A**) Representative images of histochemical GUS staining in 21-day-old *SSPP:GUS* transgenic *Arabidopsis* plants grown under well-watered (Control) and drought conditions. (**B**) Quantitative analysis of *SSPP* transcript levels determined by quantitative RT-PCR. Rosette leaves were sampled from 21-day-old wild-type (WT) plants under control and drought conditions. The *TIP41-like* gene was used as an internal control. Data are presented as mean ± SD of technical replicates (*n* = 3). Asterisk indicates significant differences compared with the control (Student’s *t*-test, *p* < 0.01). Similar expression trends were observed in at least three independent biological replicates.

To investigate whether SSPP is involved in drought stress response, we first examined its expression pattern under drought conditions using the previously described *SSPP:GUS* transgenic *Arabidopsis* [10]. The results revealed lower GUS activity in 21-day-old *SSPP:GUS* plants subjected to drought stress compared with well-watered controls (Figure 1A), suggesting that the promoter activity of *SSPP* is repressed under drought conditions. Changes in *SSPP* transcript levels in response to drought stress were also analyzed by quantitative RT-PCR. Consistently, qRT-PCR analysis showed that the *SSPP* transcript level decreased to approximately 29% of those under well-watered conditions after drought treatment (Figure 1B). These results demonstrate that the expression of *SSPP* is negatively regulated by drought stress.

### 2.2. Drought Stress Attenuates N^7^-Mediated SSPP Degradation and Confers Enhanced Drought Tolerance in N^7^-SSPP-Overexpressing Arabidopsis

**Figure 2:**
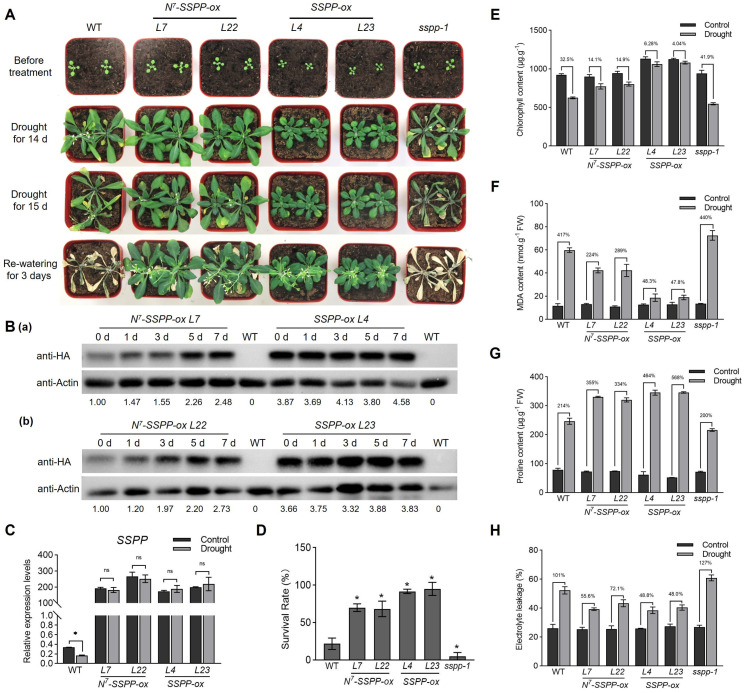
Drought stress suppresses N^7^-mediated SSPP degradation and enhances drought tolerance in *N^7^-SSPP-overexpressing Arabidopsis*: (**A**) Representative images showing the drought responses of WT, two independent *N^7^-SSPP-overexpressing* lines *(N^7^-SSPP-ox*, *L7* and *L22*), two independent *SSPP-overexpressing* lines (*SSPP-ox*, *L4* and *L23*), and *sspp-1* plants. The 14-day-old plants (imaged before treatment) were subjected to drought stress by withholding water for more than two weeks. Images were taken at 14 and 15 days after treatment (DAT). After an additional 3 days without watering, plants were re-watered and photographed 3 days after rewatering. One representative experiment from three independent biological replicates with consistent trends is shown. Survival rates were quantified as shown in (**D**). (**B**) Drought stress progressively attenuates N^7^-mediated SSPP degradation. (**a**,**b**) SSPP protein abundance examined by immunoblot analysis during drought treatment in (**a**) *N^7^-SSPP-ox* (*L7*) and *SSPP-ox* (*L4*) plants, and (**b**) *N^7^-SSPP-ox* (*L22*) and *SSPP-ox* (*L23*) plants. 21-day-old plants maintained at ~50% soil relative water content (RWC) were subjected to drought by withholding water. Rosette leaves were harvested at 0, 1, 3, 5, and 7 days after the onset of drought treatment. SSPP protein levels were detected using an anti-HA antibody, with Actin as a loading control. Numbers below the blots indicate normalized SSPP band intensities relative to the 0-day sample of *N^7^-SSPP-ox*, which was set to 1. One representative blot from three independent biological replicates with consistent trends is shown. (**C**) Transcript levels of *SSPP* were determined by quantitative RT-PCR in rosette leaves of WT and transgenic plants under control and drought conditions. The *TIP41-like* gene was used as an internal reference. Data are presented as mean ± SD of technical replicates (*n = 3*). Asterisks indicate significant differences compared with the control conditions (Student’s *t*-test, *p* < 0.01). Similar expression trends were observed in at least three independent biological replicates. (**D**) Survival rates were quantified 3 days after rewatering following drought treatment. Survival rates were calculated from independent biological replicates and analyzed across replicate means. Three independent biological replicates were performed, with 20 plants per genotype in each replicate. (**E**–**H**) Physiological responses to drought stress. Chlorophyll content (**E**), MDA content (**F**), proline content (**G**), and electrolyte leakage (**H**) were quantified in rosette leaves of WT, *N^7^-SSPP-ox*, *SSPP-ox*, and *sspp-1* plants under control and drought conditions. For chlorophyll content and electrolyte leakage measurements, leaves were harvested at 14 DAT. For MDA and proline content determination, leaves were collected when the soil RWC reached approximately 20%. Drought-induced changes are presented as percentages relative to the corresponding well-watered controls. Data in (**D**–**H**) are presented as mean ± SD from three independent biological replicates. Statistical significance was determined by Student’s *t*-test by comparing each genotype with WT. Asterisks indicate significant differences (*p* < 0.01).

To further elucidate the role of SSPP in drought tolerance, two independent transgenic lines overexpressing *SSPP* (*35S:SSPP-HA*; *SSPP-ox*, *L4* and *L23*), two lines overexpressing *N^7^-SSPP* (*35S:N^7^-SSPP-HA*; *N^7^-SSPP-ox*, *L7* and *L22*), and the *sspp-1* mutant, as described previously [10,11,12], were subjected to drought stress. After 14–15 days of drought treatment, the leaves of wild-type (WT) and *sspp-1* plants turned yellow and exhibited severe wilting, while those of *SSPP-ox* and *N^7^-SSPP-ox* plants remained largely green (Figure 2A). Consistently, drought caused a 32.5% reduction in chlorophyll content in WT and a 41.9% reduction in *sspp-1* leaves, while chlorophyll reduction in *SSPP-ox* lines was only 6.28% and 4.04%, and modest in *N^7^-SSPP-ox* plants (14.1% and 14.9%) (Figure 2E). Following rewatering for 3 days, survival rates were assessed. Only 21.7% of WT plants survived, whereas *SSPP-ox* lines displayed markedly higher survival (91.7% and 95%), and *N^7^-SSPP-ox* plants reached 70% and 68.3% (Figure 2D). In contrast, the *sspp-1* mutants almost failed to recover after rehydration (Figure 2A,D). Notably, though qRT-PCR analysis showed that the transcript levels of *SSPP* were comparable between *SSPP-ox* and *N^7^-SSPP-ox* plants and were not significantly affected by drought treatment (Figure 2C), immunoblot analysis revealed striking differences in SSPP protein accumulation. Under drought conditions, SSPP protein levels in *N^7^-SSPP-ox* (*L7* and *L22*) accumulated progressively with prolonged stress, whereas SSPP protein levels in *SSPP-ox* lines (*L4* and *L23*) remained consistently high throughout the treatment period (Figure 2B). By comparison, under control conditions, SSPP protein abundance in *N^7^-SSPP-ox* plants was markedly lower than that in *SSPP-ox* lines and remained relatively stable over time (Supplementary Figure S1). Together, these results demonstrate that although *SSPP* transcripts accumulate to similar levels, SSPP protein abundance is differentially regulated via the N^7^ element, and drought stress enhances the stability of the N^7^-SSPP fusion protein. Notably, the N^7^ element has previously been demonstrated to function as a degradation signal that targets proteins to the ubiquitin-26S proteasome pathway [14]. Therefore, the drought-induced increase in N^7^-SSPP abundance observed here is consistent with attenuation of N^7^-mediated proteasome-dependent turnover. Importantly, the N^7^ element-mediated SSPP degradation effectively rescued the growth defects observed in *SSPP-ox* lines under normal conditions (Figure 2A), as reported previously [11,12].

Drought stress is usually associated with oxidative, osmotic, and ionic stresses, which lead to cellular damage [15]. To evaluate whether SSPP enhances drought tolerance by alleviating drought-induced injury, lipid peroxidation (MDA content), proline accumulation, and electrolyte leakage (EL) were measured. Under drought conditions, *SSPP-ox* and *N^7^-SSPP-ox* plants exhibited markedly lower MDA accumulation than WT, whereas *sspp-1* showed the highest MDA level (Figure 2F). Moreover, *SSPP-ox* and *N^7^-SSPP-ox* plants accumulated significantly higher levels of proline and exhibited reduced EL compared with WT plants under drought stress, while *sspp-1* mutants showed decreased proline accumulation and increased EL (Figure 2G,H). Collectively, these findings demonstrate that drought stress stabilizes SSPP protein in *N^7^-SSPP-ox* plants, thereby enhancing drought tolerance by mitigating drought-induced cellular damage while maintaining normal growth.

### 2.3. SSPP Enhances Drought Tolerance Through Up-Regulation of ROS-Scavenging Pathways

**Figure 3:**
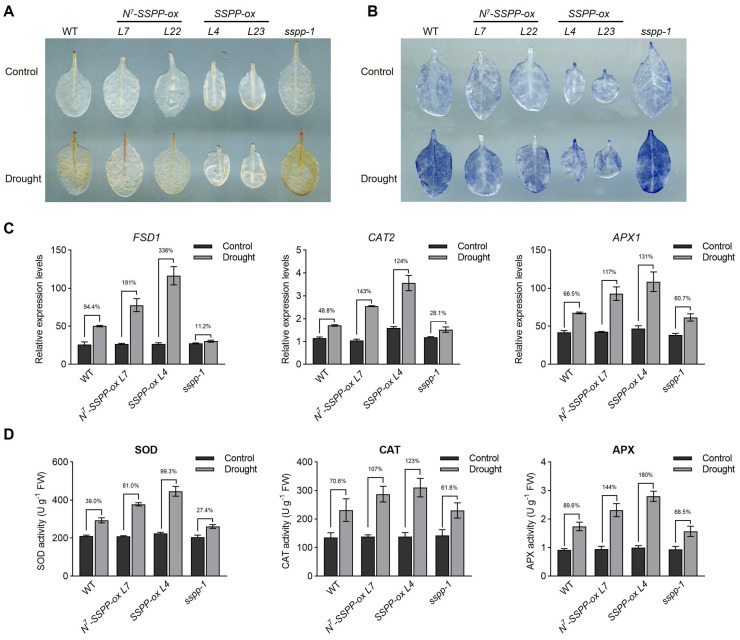
SSPP enhances antioxidant capacity and reduces drought-induced ROS accumulation in *Arabidopsis*: (**A**,**B**) Histochemical detection of H_2_O_2_ and O_2_^•−^ accumulation in detached rosette leaves by 3,3′-diaminobenzidine (DAB, dark brown) staining (**A**) and nitroblue tetrazolium (NBT, dark blue) staining (**B**), respectively. Representative images from three independent biological replicates with consistent trends are shown. (**C**) Relative transcript levels of *FSD1*, *CAT2*, and *APX1* were determined by quantitative RT-PCR in the fifth and sixth rosette leaves of WT, a representative *N^7^-SSPP-overexpressing* line (*N^7^-SSPP-ox*, *L7*), a representative *SSPP-overexpressing* line (*SSPP-ox*, *L4*), and *sspp-1*. Leaves were harvested at 10 DAT. The *TIP41-like* gene was used as an internal control. Drought-induced changes are presented as percentages relative to the corresponding well-watered controls. Data are presented as mean ± SD of technical replicates (*n* = 3). The experiment was repeated with at least three independent biological replicates, showing similar trends. (**D**) The antioxidant enzyme activities of SOD, CAT, and APX in rosette leaves of WT, *N^7^-SSPP-ox* (*L7*), *SSPP-ox* (*L4*), and *sspp-1* plants under control and drought conditions were measured. Drought-induced changes are presented as percentages relative to the corresponding well-watered controls. Data are presented as mean ± SD from three independent biological replicates.

Given the central role of ROS homeostasis in drought stress adaptation [16], we next investigated whether SSPP-mediated drought tolerance is associated with altered ROS accumulation and antioxidant capacity. To visualize and quantify the in vivo accumulation of H_2_O_2_ and O_2_^•−^, detached leaves from WT, *SSPP-ox*, *N^7^-SSPP-ox*, and the *sspp-1* mutant were stained with 3,3′-diaminobenzidine (DAB, dark brown, Figure 3A) and nitroblue tetrazolium (NBT, dark blue, Figure 3B), respectively. The results showed that although no significant differences were observed among the genotypes under normal conditions, *SSPP-ox* and *N^7^-SSPP-ox* leaves exhibited significantly lower staining intensities compared to WT and *sspp-1* leaves under drought conditions (Figure 3A,B), suggesting that overexpression of *SSPP* significantly reduced the drought-induced ROS accumulation in *Arabidopsis*. Consistent with these observations, we examined the transcriptional expression of three key ROS homeostasis-related genes, including *iron superoxide dismutase 1* (*FSD1*), *catalase 2* (*CAT2*), and *ascorbate peroxidase 1* (*APX1*), which encode crucial antioxidant enzymes involved in ROS scavenging and redox modulation [17,18,19]. Quantitative RT-PCR analysis showed that drought stress induced the expression of *FSD1*, *CAT2*, and *APX1* in the *SSPP-ox*, *N^7^-SSPP-ox* and WT plants. However, the transcript levels of all three genes were significantly higher in the transgenic lines than in the WT control (Figure 3C). In contrast, drought-induced up-regulation of these antioxidant genes was markedly attenuated in the *sspp-1* mutant (Figure 3C). In line with the transcriptional changes, drought stress significantly enhanced the enzymatic activities of superoxide dismutase (SOD), catalase (CAT), and ascorbate peroxidase (APX) in *SSPP-ox* and *N^7^-SSPP-ox* plants compared with WT, whereas *sspp-1* mutants displayed much lower enzyme activities under the same conditions (Figure 3D). Together, these results indicate that SSPP positively regulates antioxidant defense at both transcriptional and enzymatic levels, thereby promoting the maintenance of ROS homeostasis and enhancing drought tolerance in *Arabidopsis*.

Considering that SSPP has also been implicated in the regulation of senescence [10], we further examined whether the enhanced drought survival of *N^7^-SSPP-ox* and *SSPP-ox* plants is associated with altered senescence progression, altered water status, or a combination of both. To this end, we analyzed the expression of a senescence-associated gene, *AtSARK*, a senescence-upregulated receptor-like kinase that functions antagonistically with the phosphatase SSPP in regulating senescence progression [20]. Under drought conditions, *AtSARK* was strongly induced in WT plants, whereas the induction was markedly attenuated in both *N^7^-SSPP-ox* and *SSPP-ox* lines (Supplementary Figure S2A). By contrast, the *sspp-1* mutant exhibited enhanced induction of *AtSARK* in response to drought stress (Supplementary Figure S2A), suggesting that SSPP negatively regulates drought-triggered senescence progression. In parallel, detached-leaf water-loss assays were conducted to assess whether SSPP also influences leaf water status under drought conditions. The results showed that no significant differences in water-loss rates between *SSPP-ox*, *N^7^-SSPP-ox* plants and WT, suggesting that the improved drought performance is mainly attributable to enhanced drought tolerance (Supplementary Figure S2B). Collectively, together with the reduced ROS accumulation and elevated antioxidant capacity observed in *SSPP-overexpressing* plants (Figure 3), these findings support a model in which SSPP improves drought performance primarily through oxidative stress mitigation and delayed senescence.

## 3. Discussion

### 3.1. SSPP Integrates Senescence Regulation with Drought Stress Adaptation

Drought stress is a major abiotic constraint on plant growth and productivity, typically causing osmotic imbalance, excessive ROS accumulation, and premature leaf senescence [5,6]. In this study, we demonstrate that SSPP positively regulates drought tolerance in *Arabidopsis*, extending its previously reported roles in senescence delay and salt stress response to include drought conditions. Importantly, our findings reveal a counterintuitive pattern in which *SSPP* transcription is repressed under drought stress (Figure 1), whereas SSPP protein accumulation is enhanced, particularly in the N^7^-SSPP fusion context (Figure 2B). Such an inverse relationship between transcript abundance and protein accumulation highlights a dual-layer regulatory strategy, in which transcriptional repression is coupled with post-translational stabilization. This strategy may allow plants to limit energy-consuming transcriptional programs during prolonged stress while selectively maintaining proteins that confer stress protection. Our results therefore support the idea that post-translational control plays a crucial role in fine-tuning stress adaptation, beyond transcriptional regulation alone.

Both *SSPP-overexpressing* and *N^7^-SSPP-overexpressing* plants exhibited enhanced drought tolerance, as evidenced by reduced leaf chlorosis, higher survival rates after rewatering, and attenuated cellular damage, whereas the *sspp-1* mutant displayed pronounced drought stress sensitivity (Figure 2). Together with earlier studies demonstrating that SSPP delays senescence and enhances salt tolerance [10,11], these data position SSPP as an important regulatory node at the intersection of senescence progression and abiotic stress adaptation. Nevertheless, while the present study establishes a functional role for SSPP in drought tolerance, the molecular pathways downstream of *SSPP* under drought stress remain to be fully elucidated.

### 3.2. SSPP Enhances Drought Tolerance by Reinforcing Antioxidant Capacity

Maintaining ROS homeostasis is essential for plant survival under drought conditions [21]. Our results show that SSPP positively regulates the expression of several key ROS-scavenging genes, including *FSD1*, *CAT2*, and *APX1*, and enhances the activities of their corresponding antioxidant enzymes (Figure 3C,D). These transcriptional and biochemical changes are accompanied by reduced ROS accumulation (Figure 3A,B), lower membrane damage, increased proline accumulation, and decreased EL (Figure 2F–H) in *SSPP-ox* and *N^7^-SSPP-ox* plants under drought stress. Moreover, detached-leaf water loss assays revealed no significant differences in water loss rates among genotypes (Supplementary Figure S2B), indicating that the enhanced drought tolerance of *SSPP-overexpressing* plants is unlikely to result from altered water status or drought avoidance. Instead, the data support a model in which SSPP primarily enhances drought tolerance through improved cellular protection, including strengthened antioxidant capacity and delayed senescence progression.

SSPP belongs to the PP2C family of protein phosphatases, members of which are well known for their roles in abscisic acid (ABA) signaling and stress responses. While clade A PP2Cs typically function as negative regulators of ABA signaling [22], SSPP appears to play a distinct role as a positive regulator of stress tolerance. Although the direct substrates of SSPP under drought stress remain unknown, it is conceivable that SSPP may target components involved in ROS signaling, senescence-associated transcriptional networks, or MAPK cascades. Identification of these substrates will be an important direction for future studies aimed at placing SSPP within established drought signaling pathways.

### 3.3. Drought Stress Modulates N^7^-Mediated SSPP Protein Turnover

Constitutive overexpression of *SSPP* enhances stress tolerance but also leads to growth inhibition, highlighting the need for regulatory mechanisms that uncouple stress protection from growth penalties. The N^7^ degron provides such a mechanism by enabling conditional control of SSPP protein stability [11]. Under normal conditions, the N^7^-SSPP protein remained at low levels, and plant growth was comparable to that of the WT plants (Figure 2A and Figure S1). However, drought stress induces a progressive accumulation of N^7^-SSPP protein without altering *SSPP* transcript levels (Figure 2B,C). The N^7^ element has previously been shown to confer ubiquitin-26S proteasome-dependent degradation of fusion proteins [14]. Within this established mechanistic framework, the drought-induced accumulation of N^7^-SSPP observed here is consistent with attenuation of N^7^-mediated protein turnover, rather than enhanced transcription or altered protein extraction efficiency.

Although the precise molecular signals that inhibit N^7^-dependent degradation under drought remain unclear, it is plausible that diverse stress conditions converge on shared downstream processes, such as redox imbalance or stress-activated kinases, that influence proteasome-mediated protein turnover. Elucidating how drought signals intersect with the N^7^ degradation pathway will provide deeper insight into how plants dynamically regulate protein stability in response to fluctuating environments.

### 3.4. N^7^-SSPP Fusion Provides a Strategy to Balance Growth and Stress Resistance

The N^7^-SSPP fusion strategy effectively separates the beneficial effects of SSPP on drought tolerance from its inhibitory effects on growth. Consistent with our previous quantitative analyses under non-stress conditions [11], *N^7^-SSPP-overexpressing* plants exhibit growth characteristics comparable to WT plants, in contrast to the growth inhibition observed in constitutive *SSPP-overexpressing* lines. In the present study, *N^7^-SSPP-overexpressing* plants maintained near-normal growth under control conditions while exhibiting significantly enhanced drought tolerance (Figure 2), underscoring the potential of conditional protein stabilization as a tool for stress resilience.

While these findings highlight the promise of N^7^-mediated regulation for crop improvement, several limitations should be considered. The behavior of the N^7^ element may vary across species, and drought conditions in the field are often more heterogeneous than controlled laboratory settings. In addition, constitutive promoters such as *CaMV 35S* may not provide optimal spatial or temporal control of transgene expression in crops. Future efforts should therefore focus on testing the N^7^ element in crop species and combining it with stress-inducible or tissue-specific promoters to maximize agronomic relevance.

## 4. Methods and Materials

### 4.1. Plant Materials and Growth Conditions

All *Arabidopsis thaliana* materials used in this study are in the Columbia (Col-0) background. The genetic materials *SSPP:GUS*, *35S:SSPP-HA*, *35S:N^7^-SSPP-HA*, and *sspp-1* have been described previously [10,11,12]. *Arabidopsis* seeds were sterilized in 1% (*v*/*v*) sodium hypochlorite, washed with sterilized water, and germinated on half-strength Murashige and Skoog (MS) medium (0.8% [*w*/*v*] agar, pH 5.7, 1% [*w*/*v*] sucrose) in a plant growth chamber (Percival Scientific, Perry, IA, USA) at 22/19 °C with cycles of 16 h light and 8 h darkness under 100–150 μmol m^−2^ s^−1^ light intensity. The 7-day-old seedlings were then transferred to peat soil and grown under the same conditions for further experiments.

### 4.2. Soil-Drying Drought Treatment and Survival Assay

Drought stress in this study refers to soil-drying drought imposed by withholding water. Drought treatments were performed with minor modifications based on a previously described method [23]. The 7-day-old seedlings were transferred to pots containing an equal dry mass of peat-based soil mixture (30 g per pot) and grown in the greenhouse for an additional week. Drought treatment was initiated at 14 d after seedlings emerged by withholding water. To minimize positional effects, pots were randomized and rotated daily throughout the treatment period.

To ensure comparable drought progression among genotypes that may differ in transpiration, sampling and rewatering were standardized based on soil relative water content (RWC) rather than on time alone. Soil RWC was monitored daily by pot weighing, and drought progression curves for each genotype are provided (Supplementary Figure S3). Rosette leaves were harvested for chlorophyll content and electrolyte leakage measurements when the soil RWC decreased below ~15% (approximately 14 DAT). Plants were rewatered when the soil RWC reached ~5% (approximately 18 DAT), and survival rates were assessed 3 days after rewatering. Plants were scored as survivors when new leaf growth resumed. Survival rate assays were performed using three independent biological replicates, each replicate containing at least 20 plants per genotype.

### 4.3. Detached Leaf Water Loss Assay

For detached-leaf water loss assays, fully expanded rosette leaves were detached from 4-week-old plants grown under well-watered conditions. Leaves were immediately placed on weighing paper at room temperature, and fresh weight was recorded at 10 min intervals. Water loss was calculated as the percentage of initial fresh weight. The experiment was repeated at least three times independently.

### 4.4. RNA Extraction and Quantitative Real-Time PCR Analyses

Total RNA was extracted with an RNA Easy Fast Plant Tissue Kit (TIANGEN, Beijing, China) and reverse-transcribed with a HiScript II Q RT SuperMix (Vazyme, Nanjing, China) according to the manufacturer’s instructions. Quantitative RT-PCR was performed on a CFX Duet Real-Time PCR Supplemental System (BIO-RAD, Hercules, CA, USA) using TB Green Premix Ex Taq™ II (Takara, Dalian, China). The *TIP41-like* gene was used as an internal control.

All primer sequences, amplification efficiencies, and melt curve validation are provided in Supplementary Table S1. Primer efficiencies were within an acceptable range, and single peaks in melt curve analyses confirmed amplification specificity.

### 4.5. Histochemical GUS Staining and ROS Detection

Histochemical GUS staining was performed as described previously [24]. For ROS detection, detached leaves were stained with DAB or NBT. For DAB staining, leaves were incubated in DAB solution (1 mg/mL DAB, 50 mM MES, pH 3.8, 0.05% Tween 20) for 8 h in the dark. For NBT staining, leaves were incubated in NBT solution (0.1 mg/mL NBT, 50 mM Tris-HCl, pH 7.5, 0.05% Tween 20) for 4 h in the dark. Leaves were subsequently decolorized in bleaching solution (ethanol:acetic acid:glycerol = 3:1:1, *v*/*v*/*v*). Stained samples were imaged using a flatbed scanner (EPSON, Suwa, Japan).

### 4.6. Protein Extraction and Immunoblot Analysis

Total soluble proteins were extracted from rosette leaves as described previously [25]. Western blot analysis was performed using rabbit anti-HA (Abcam, Cambridge, UK) and mouse anti-Actin (Abmart, Shanghai, China) antibodies [12]. Protein band intensities were quantified using ImageJ software, version 1.49v with background subtraction. The Western blot analysis for the drought time course was performed with three independent biological replicates to ensure reproducibility.

### 4.7. Measurements of Chlorophyll, MDA, Proline, and Electrolyte Leakage

The chlorophyll content in the leaves of *Arabidopsis* was spectrophotometrically determined according to Arnon (1949) [26].

MDA and proline contents were determined using commercial assay kits (Solarbio, Beijing, China) according to the manufacturers’ instructions.

Electrolyte leakage was measured as described previously with minor modifications [27]. Briefly, rosette leaves were washed thoroughly with running distilled water and incubated in fresh distilled water. The initial electrical conductivity (C0) was measured using a conductivity meter, followed by incubation at 40 °C for 30 min to obtain conductivity (C1). Subsequently, samples were boiled at 100 °C for 10 min to obtain conductivity (C2). Electrolyte leakage was calculated as follows: EL (%)=[(C1−C0)/(C2−C0)]×100.

### 4.8. Antioxidant Enzyme Activity Assays

The activities of SOD, CAT, and APX were determined using commercial assay kits (Solarbio, Beijing, China) following the manufacturers’ instructions.

### 4.9. Determination of Soil Relative Water Content (RWC)

Soil relative water content (RWC) was determined by pot weighing. *Arabidopsis* plants were grown in pots containing 30 g dry soil mass, and all pots were saturated with water prior to sowing to ensure identical initial soil moisture conditions. Pots were fully saturated by subirrigation and weighed to obtain turgid weight (TW). During drought treatment, pot fresh weight (FW) was recorded daily. Soil RWC was calculated as: RWC (%)=[(FW−DW)/(TW−DW)]×100, where DW represents the dry weight of the soil mixture. Drought progression curves were determined for each genotype and are shown in Supplementary Figure S3.

Based on soil RWC measurements, different physiological and molecular analyses were performed at defined drought stages. *SSPP:GUS* and WT plants maintained under moderate soil moisture conditions (~50% soil RWC, Control) or subjected to drought conditions (~30% soil RWC) were sampled for histochemical GUS staining and qRT-PCR analyses. For drought time-course experiments, transgenic plants grown under moderate soil moisture (~50% soil RWC) were subjected to soil-drying drought by withholding water, and rosette leaves were harvested at 0, 1, 3, 5, and 7 days after drought initiation for protein extraction and immunoblot analysis. For MDA and proline quantification, all *Arabidopsis* plants were subjected to drought stress until soil RWC reached approximately 20%, and the fifth and sixth rosette leaves were harvested for analysis.

## 5. Conclusions

In summary, our study identifies a novel biological function for SSPP in conferring drought tolerance in *Arabidopsis*. SSPP enhanced drought performance by reinforcing antioxidant defense capacity, reducing ROS accumulation, and alleviating drought-induced cellular damage. We further demonstrate that drought stress suppresses N^7^-mediated SSPP degradation, enabling conditional accumulation of SSPP protein in *N^7^-SSPP-overexpressing* plants. Importantly, the N^7^-SSPP fusion strategy retains drought tolerance benefits while avoiding growth inhibition under normal conditions. These findings advance our understanding of post-translational regulation during drought stress adaptation and highlight the potential of N^7^-mediated protein stability control as a useful strategy for improving crop stress resilience.

## Data Availability

Data are contained within the article and Supplementary Materials.

## Supplementary Materials

The following supporting information can be downloaded at https://www.mdpi.com/article/10.3390/ijms27062651/s1.
