# Supplementary material for: N7–SSPP Confers Drought Tolerance in Arabidopsis
Source: Int J Mol Sci. 2026 Mar 13;27(6):2651. doi: 10.3390/ijms27062651 (PMC13027292; doi:10.3390/ijms27062651)
Supplement: Supplementary file 1 [file ijms-27-02651-s001.zip › Supplementary files/Supplementary Figure S1.pdf]

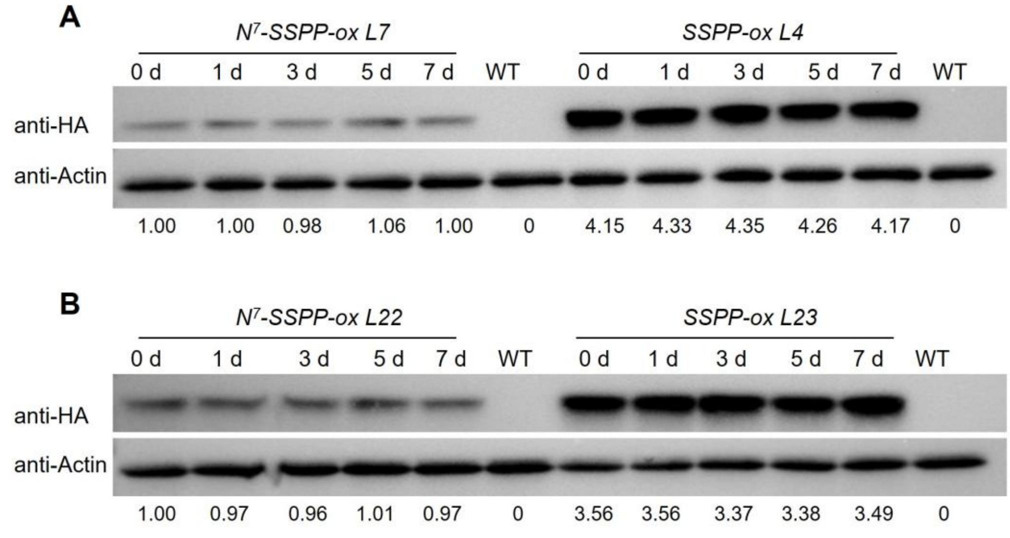


**Supplementary Figure S1**. $N^7$-mediated SSPP degradation under control conditions: (**A,B**) SSPP protein abundance in two independent *$N^7$-SSPP-overexpressing* lines (*$N^7$-SSPP-ox*, *L7* and *L22*) and two independent *SSPP-overexpressing* lines (*SSPP-ox*, *L4* and *L23*) under well-watered (control) conditions. Rosette leaves from 21-day-old plants maintained at ~50% soil RWC were harvested at 0, 1, 3, 5, and 7 days after mock treatment. SSPP protein levels were detected using an anti-HA antibody, with Actin serving as a loading control. Numbers below the blots indicate normalized SSPP band intensities relative to the 0-day sample of *$N^7$-SSPP-ox*, which was set to 1. WT plants were included as a negative control. One representative blot from three independent biological replicates with consistent trends was shown.
