# Supplementary material for: N7–SSPP Confers Drought Tolerance in Arabidopsis
Source: Int J Mol Sci. 2026 Mar 13;27(6):2651. doi: 10.3390/ijms27062651 (PMC13027292; doi:10.3390/ijms27062651)
Supplement: Supplementary file 1 [file ijms-27-02651-s001.zip › Supplementary files/Supplementary Figure S2.pdf]

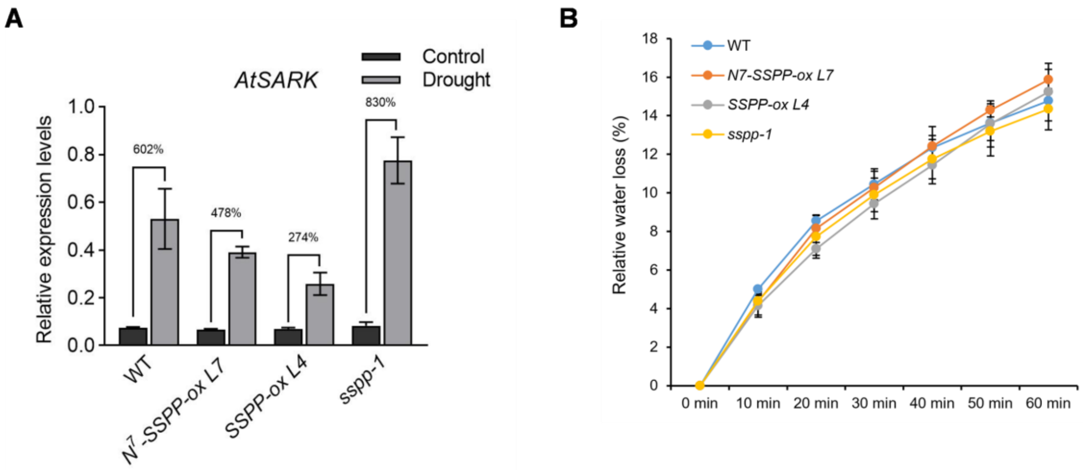

Supplementary Figure S2. SSPP modulates drought-induced senescence progression but does not affect water-loss rates: (**A**) Relative transcript levels of the senescence-associated gene *AtSARK* in rosette leaves of WT, *N^7-SSPP-ox* (*L7*), *SSPP-ox* (*L4*), and *sspp-1* plants under control and drought conditions. The *TIP41-like* gene was used as an internal reference. Drought-induced changes are presented relative to the corresponding well-watered controls. Data are presented as mean ± SD of technical replicates (n = 3). Similar expression trends were observed in at least three independent biological replicates. (**B**) Detached-leaf water-loss assay. Fully expanded rosette leaves from 4-week-old plants grown under well-watered conditions were detached and weighed at the indicated time points. Water loss is expressed as the percentage of initial fresh weight. Data represent mean ± SD from three independent biological replicates.
