# Supplementary material for: N7–SSPP Confers Drought Tolerance in Arabidopsis
Source: Int J Mol Sci. 2026 Mar 13;27(6):2651. doi: 10.3390/ijms27062651 (PMC13027292; doi:10.3390/ijms27062651)
Supplement: Supplementary file 1 [file ijms-27-02651-s001.zip › Supplementary files/Supplementary Figure S3.pdf]

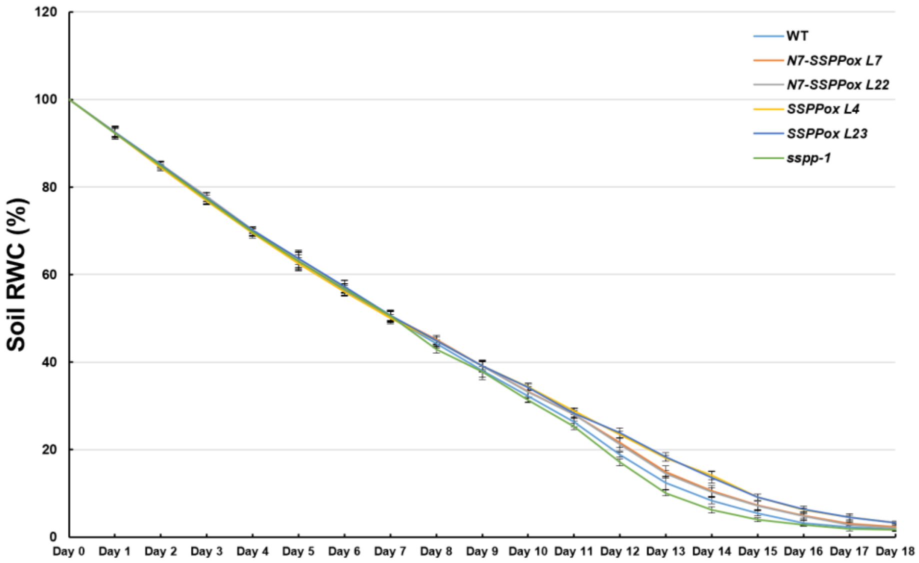

Supplementary Figure S3. Soil relative water content dynamics during drought treatment: Soil RWC progression curves for WT, N^7-*SSPP-ox* (*L7* and *L22*), *SSPP-ox* (*L4* and *L23*), and *sspp-1* plants during soil-drying drought treatment. Pots containing equal dry soil mass (30 g per pot) were fully saturated prior to drought initiation. Soil RWC was monitored daily by pot weighing and calculated as described in the Methods. Data represent mean ± SD from three independent biological replicates.
